# Oral health professionals must use the correct terminology when explaining risks for complications and undesirable health outcomes as a basis for informed consent for clinical treatment

**DOI:** 10.1002/cre2.237

**Published:** 2019-08-21

**Authors:** Asbjorn Jokstad

1

Patient care stakeholders make use of different terms when disputing clinical treatment that has not evolved as planned or anticipated. Some favor terms as a reflection of their educational background or due to legal considerations or simply because of ignorance of the exact meaning of terms without recognizing that words such as “complication,” “adverse outcome,” and “treatment failure” are highly ambiguous and individually biased. Lewis Carroll exposes the fallacies of endowing words in common usage with particular meanings in his amusing book titled “*Through the Looking Glass*”: “‘When I use a word,’ Humpty Dumpty said, in rather a scornful tone, ‘it means just what I choose it to mean—neither more nor less.’ ‘The question is,’ said Alice, ‘whether you can make words mean so many different things.’” (Carroll, 1872).

Examples of ambiguous communication abound in dentistry, perhaps because several textbooks fail to define the term or offer odd definitions. The term “complication” has never appeared in the authoritative “Glossary of prosthodontic terms” and the latest edition defines “failure” as “the inability of a prosthesis to produce the expected desired outcome” (ACP, 2017). Another authoritative source, that is, The glossary of oral and maxillofacial implants, endorsed by the ITI International Team for Implantology, propose that a complication is “an unexpected deviation from a normal treatment outcome” (Laney, 2007). However, “normal treatment outcome” is a figment of imagination because a treatment outcome depends on a range of factors and interindividual premises and “normal” is an indefinite word. Moreover, “deviation from” may also signify that an outcome may be, in terms of use of adjectives, “perfect,” which contextually is better than “normal,” alternatively, in terms of statistical thinking, superior to the treatment that half the patient population experienced. A third authoritative source suggests that a complication “… is a difficulty resulting from single or multiple factors that demand additional clinical interventions” (Zarb, Hobkirk, Eckert, & Jacob, 2013). The erraticism in the dental literature can also be identified by searching Pubmed using merely the phrase “complication*[ti]” and filtered for dental journals (*n* = 2,867 papers). It is striking that many scientific articles summarize clinical data in a way that reflects a perception that ill health, or potential for ill health, associated with a dental device, such as an implant, endodontic sealer, restoration, or prosthesis, is a “complication” if the condition is corrigible and a “failure” when the dental device is non‐amendable and requires removal and replacement or remake.

The examples above demonstrate use of terminology that is not in alignment with contemporary terminology used in context with patient safety and patient rights. The oral health research and clinician communities should therefore reconsider current use of terminology, especially in light of the extensive activities worldwide over the last decade to promote patient safety in health care under the auspice of WHO (WHO, 2019). A significant component of this effort was to define precise terms related to patient safety as a basis for an international taxonomy while discouraging the use of ambiguous and depreciated terms and words that have variable connotations amongst different jurisdictions (e.g., “negligence”). The most recent International Classification for Patient Safety Key Concepts and Preferred Terms (WHO, 2019) describe an ambition that all concepts and terms in ICPS should be applicable across the full spectrum of health care from primary to highly specialized care.

It is outside of the scope of this short editorial to elaborate on how the broad range of terms and concepts in ICPS apply to scenarios in clinical oral and dental medicine, but some knowledge of a few core notions may be helpful. One umbrella term is “harmful incident,” which is synonymous to “adverse event” and is defined as any incident that result in harm to a patient. “Adverse event” relate closely with a range of other precisely defined terms (Table 1) and is defined as a “medical error” if it could have been prevented given the current state of medical knowledge. This term again is different from what constitutes “medical injury” and is not synonymous with “medical malpractice.” Another core term is “adverse patient occurrence” that encompasses a variety of situations of which many may transpire during the provision of oral health care. For example, when the patient has to be retreated because of complications or incomplete care previously; or, if there are deficiencies in documentation, such as informed consent procedures or in the medical record; or, if the procedures were employed that did not meet the criteria for appropriateness according to good oral medical practice (WHO, 2019).

**Table 1:** Harmful incident, alternatively named adverse event, relates closely with a range of other precisely defined terms in the ICPS (WHO, 2019)

Accident	Adverse drug event (ADE)	Adverse drug reaction (ADR)
Adverse patient occurrence (APO)	Adverse reaction	Adverse serious event
Bad outcome	Clinical incident	Close call
Critical incident	Dangerous situation	Drug misadventure
Error	Event	Harm
Hazard	Iatrogenic	Incident
Injury (bodily)	Life‐threatening adverse drug experience	Medical error
Medical injury	Medical mishap	Medical mistake
Medication error	Misadventure	Mistake
Near miss	No harm event	Patient safety
Patient safety incident	Potential adverse drug event	Potential adverse event
Potential error/event	Preparation error	Prescribing error
Preventable adverse drug event	Preventable adverse event	Preventable event
Preventable death	Reportable occurrence	Sentinel event
Serious event	Serious outcome	Slip
Unexpected adverse drug experience	Unpreventable adverse drug event	Unpreventable adverse event

One idiosyncrasy in the ICPS is that the term “patient health outcome” refers to the effects of activities carried out by health care providers, while “patient outcome” designate the impact upon a patient that is wholly or partially attributable to a harmful incident. The impact may be described by a pathophysiology condition or by the type of injury in combination with the degree of harm ranging from none, mild, moderate, severe to death according to the specific criteria, and furthermore combined with social and/or economic impact.

Another idiosyncrasy of the ICPS is that “complications” include three definitions; of which one apply only to hospital settings, that is, “a diagnosis occurring during hospitalization that is thought to extend the hospital stay at least one day for roughly 75% or more of the patients.” The two remaining definitions, however, focus on events happening during or immediately following the provision of care, that is, “a detrimental patient condition that arises during the process of providing health care, regardless of the setting in which the care is provided” and “a disease or injury that arises subsequent to another disease and/or health‐care intervention.” These two definitions are in alignment with the Medline MESH definition (“conditions that co‐exist or follow, i.e., co‐existing diseases, complications, or sequelae”) and dictionary definitions. For example, Webster's dictionary defines medical complications as “A disease or diseases, or adventitious circumstances or conditions, coexistent with and <negatively> modifying a primary disease, but not necessarily connected with it,” while the Oxford dictionary proposes: “A secondary disease or condition aggravating an already existing one.”

**Figure 1:**
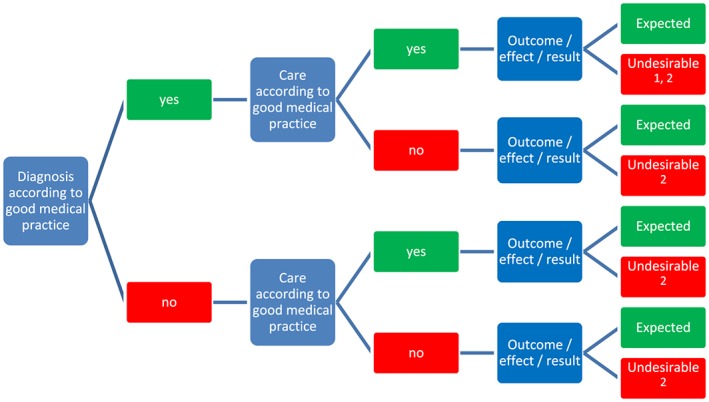
Relationship between a medical complication (1) and an undesirable patient health outcome (2) following provision of health care. In some countries, “good medical practice” is termed “standard of care.” “Good medical practice” is secondary to a range of socioeconomic and political determinants that includes health system organization and infrastructure, availability of expertise, costs, equity and access to care, amongst others

Many providers and laypersons have particular difficulties differentiating between a medical complication and an undesirable patient health outcome (Figure 1). An undesirable patient health outcome is often a consequence of a harmful incident or an inadequate diagnosis or provision of care according to good medical practice and is, therefore, in theory, preventable. A medical complication is also an undesirable patient health outcome, although their potential occurrence cannot be predicted precisely, even if the provision of care is excellent. An alternative, perhaps more technical explanation for some, is that a medical complication is essentially a stochastic phenomenon.

It is essential to realize that the global quest to improve patient safety and patient rights connects closely with quality of health care, which mandates that care provider delivers safe and effective patient‐centered care that is equitable and both timely and efficient. Excellent communication before, during, and following care is an integral component of good medical practice, and the health care provider must use the correct terminology when discussing aspects of risks and safety with patients to avoid misunderstanding.
